# Treatment options for gastrointestinal bleeding blue rubber bleb nevus syndrome: Systematic review

**DOI:** 10.1111/den.14564

**Published:** 2023-05-09

**Authors:** Alessandro Rimondi, Andrea Sorge, Alberto Murino, Nicoletta Nandi, Lucia Scaramella, Maurizio Vecchi, Gian Eugenio Tontini, Luca Elli

**Affiliations:** ^1^ Department of Pathophysiology and Transplantation University of Milan Milan Italy; ^2^ Gastroenterology and Endoscopy Unit Foundation IRCCS Ca' Granda Ospedale Maggiore Policlinico Milan Italy; ^3^ Royal Free Unit for Endoscopy The Royal Free Hospital and University College London Institute for Liver and Digestive Health London UK; ^4^ Department of Gastroenterology Cleveland Clinic London London UK

**Keywords:** endoscopy, enteroscopy, small bowel, vascular malformation

## Abstract

**Objectives:**

Blue rubber bleb nevus syndrome (BRBNS) is a rare challenging cause of gastrointestinal bleeding. We performed a systematic review of case reports and case series on BRBNS to gather information on the treatment options currently available.

**Methods:**

All studies reporting a case of BRBNS in humans were evaluated. Papers were ruled out if CARE criteria and explanations on patient's selection, ascertainment, causality, and reporting were not respected or identified. PROSPERO 2021 CRD 42021286982.

**Results:**

Blue rubber bleb nevus syndrome was treated in 106 cases from 76 reports. 57.5% of the population was under 18 years old, and up to 50% of the cases reported a previous treatment. Clinical success was achieved in 98 patients (92.4%). Three main types of interventions were identified: systemic drug therapy, endoscopy, and surgery. After BRBNS recurrence or previous therapy failure, systemic drug therapy emerged as a preferred second‐line treatment over endoscopy (*P* = 0.01), but with a higher rate of reported adverse events when compared with surgery and endoscopy (*P* < 0.001). Endoscopic treatment was associated with a higher number of required sessions to achieve complete eradication when compared with surgery (*P* < 0.001). No differences between the three main areas were found in the overall follow‐up time (*P* = 0.19) or in the recurrence rate (*P* = 0.45).

**Conclusion:**

Endoscopy, surgery, and systemic drug therapy are feasible treatment options for BRBNS. Systemic drug therapy was the favorite second‐line treatment after endoscopic failure or recurrence of BRBNS, but adverse events were more frequently reported.

## INTRODUCTION

**Figure 1:**
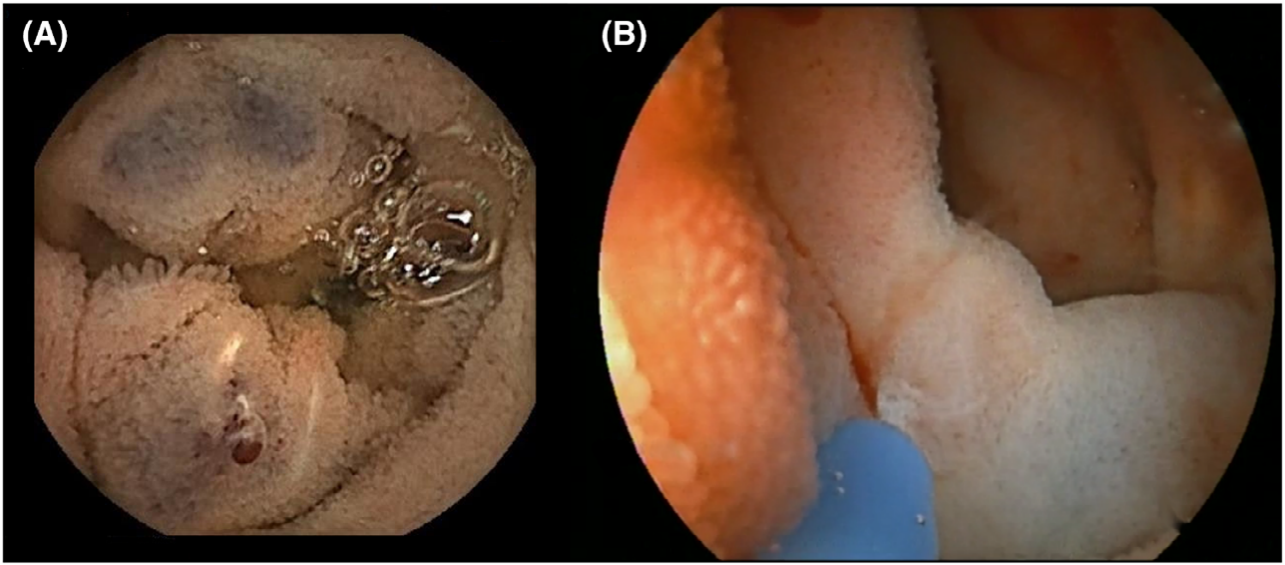
(A) Video capsule endoscopy of blue rubber bleb nevus in the jejunum. (B) The same patient being treated for bleeding blue rubber bleb nevus in the jejunum with argon plasma coagulation.

Blue rubber bleb nevus syndrome (BRBNS) is a rare disease characterized by multifocal venous malformations (VMs) that mainly involve the skin and gastrointestinal (GI) tract, although it can potentially affect any organ.^1^, ^2^ VMs located in the GI tract appear as soft, blue to purple nodules; they frequently lead to chronic bleeding, iron deficiency anemia, and/or acute hemorrhage^3^, ^4^, ^5^ (Fig. 1). Although aberrant VMs are often congenital and their size and number increase with age, VMs of BRBNS may develop in adult and elderly patients.^6^, ^7^, ^8^ Intestinal volvulus, infarction, or intussusception are infrequent complications of GI tract involvement.^9^ To date, there is no evidence of a potential malignant evolution of VMs.

The majority of BRBNS are sporadic, although rare familial clusters are reported.^10^, ^11^ Soblet *et al*.^12^ shed light on the pathogenesis of this syndrome. A double (cis) somatic mutation in *TEK*, a gene encoding for *TIE2* (angiopoietin‐tyrosine‐kinase receptor), was found in the majority of patients with BRBNS and is thought to be responsible for clinical manifestations.^12^ Although these findings need confirmation, mutations in *TIE2* can cause endothelial cell proliferation and nevus formation through the constitutive activation of the mammalian target of the rapamycin (mTOR) pathway.

Different surgical and endoscopic techniques as well as pharmacological therapies have been proposed for the treatment of GI BRBNS.^13^, ^14^, ^15^ In addition to the treatment of the bleeding lesions, lifelong support with iron infusions and blood transfusions is often needed in patients with multiple lesions and different GI segments involved.

Nowadays, most of the available evidence related to BRBNS comes from case reports and small case series;^15^, ^16^ this is due to the rarity of this syndrome, but also to the difficulties and associated delays to achieve a final diagnosis. Data from prospective studies are scant and no study comparing treatment options for BRBNS has been reported so far. Therefore, the true incidence of this syndrome is currently unknown.

To overcome these limitations, we conducted a systematic review of case reports and case series available in the literature, assessing the type of treatments available for patients affected by GI bleeding BRBNS‐related.^17^ The primary aim of our research was to clarify the success rate of the medical, endoscopic, and surgical treatments and to establish if there was any significant difference in terms of baseline characteristics and disease‐related outcomes.

## METHODS

### Study selection

We conducted a systematic review of studies, case series, and case reports in the literature following the principles of the Joanna Briggs Institute Reviewers' Manual.^18^ We searched the following terms: “blue,” “rubber,” “bleb,” and “syndrome” on the Embase, MEDLINE, and Cochrane databases up to 11 November 2021, looking for articles answering the specific question: “What kind of treatments are available for patients with bleeding gastrointestinal BRBNS?” To reduce any possible retrieval bias, additional articles were identified by searching the reference lists of the included studies. Rayyan software was employed for data collection and selection.^19^

### Inclusion and exclusion criteria

Inclusion criteria involved any publication where treatment of BRBNS GI bleeding was described. Nominal data of the patient were included, in line with the CARE criteria.^20^ In addition, only publications written in English were considered. Two authors (A.R. and A.S.) searched the online databases, ruled in papers according to the title and abstract, and then screened full‐text articles for eligibility. Disputes between the two authors were resolved through discussion and the help of a third reviewer (N.N.). For each article, we recorded the study design, the year, and the nation in which the procedures were performed. For each patient included in the study, we recorded a set of clinical variables including sex, age, age at diagnosis, the extent of GI involvement, previous treatment, the treatment described in the paper, the number of sessions needed—in case of endoscopic or surgical treatment, clinical success (defined either as reduced need of transfusions or increased hemoglobin levels), follow‐up time, adverse event (AE), recurrence, and death. A formal evaluation of four criteria (i.e. selection, ascertainment, causality, and reporting standards) of each article was performed to rule out papers that lacked significant and important clinical information. We considered a threshold of a minimum of three out of four criteria to rule in significant articles. Considering the nature of the studies, the risk of selection bias was not amendable, but it was considered in the final report. The results of our research were displayed according to PRISMA guidelines.^21^ This systematic review was registered on the PROSPERO database (PROSPERO 2021 CRD 42021286982).

### General considerations about this study methodology

**Figure 2:**
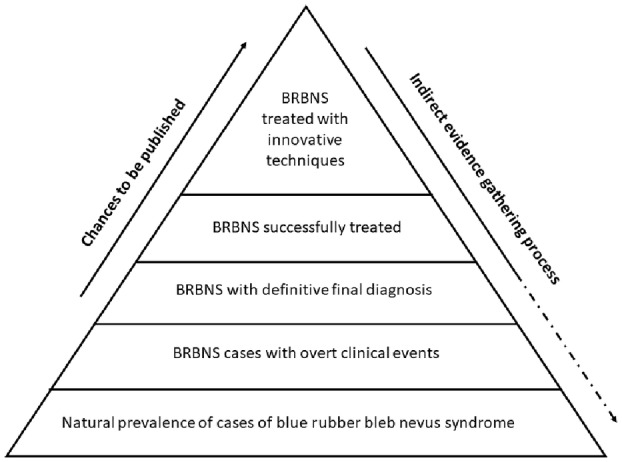
Proposed scheme for representing actual evidence regarding blue rubber bleb nevus syndrome (BRBNS) and mechanism of evidence gathering.

It was assumed that, given the rarity of this syndrome, information was to be collected mainly from case series and case reports. However, these kinds of articles present publication bias (Fig. 2). To overcome this bias, we identified three main macro intervention populations, namely, endoscopic, surgical, and pharmacological treatment. To note, when analyzing the outcome of endoscopic‐assisted surgery interventions, we counted them as part of the surgical macro‐area, considering the overall burden of the operation. This decision was also made to preserve an adequate number of observations in the three main groups, to make statistical analysis less prone to bias. The population of each macro area of intervention was determined by the number of patients treated that have been published in the literature. Our process of evidence gathering is indirect, moving from what has been reported to an estimation of the true frequency of clinical characteristics, successful treatments, and outcomes of patients with BRBNS (Fig. 2).

### Statistical analyses

Data are presented in terms of numerical variables (mean ± SD or median—interquartile range [IQR] whether a normal distribution was assumed or not) as well as categorical variables (percentage). We employed the Kruskal–Wallis one‐way ANOVA for numerical variables, as well as the χ^2^‐test with Bonferroni correction for multiple comparisons for categorical variables. For comparison between two groups, the Mann–Whitney *U‐*test and Fisher's exact test were employed in case of respectively numerical variables or categorical variables.

R Studio v. 4.0.0 (R Core Team, Vienna, Austria, https://www.R‐project.org/) was used for quantitative analyses.

## RESULTS

**Figure 3:**
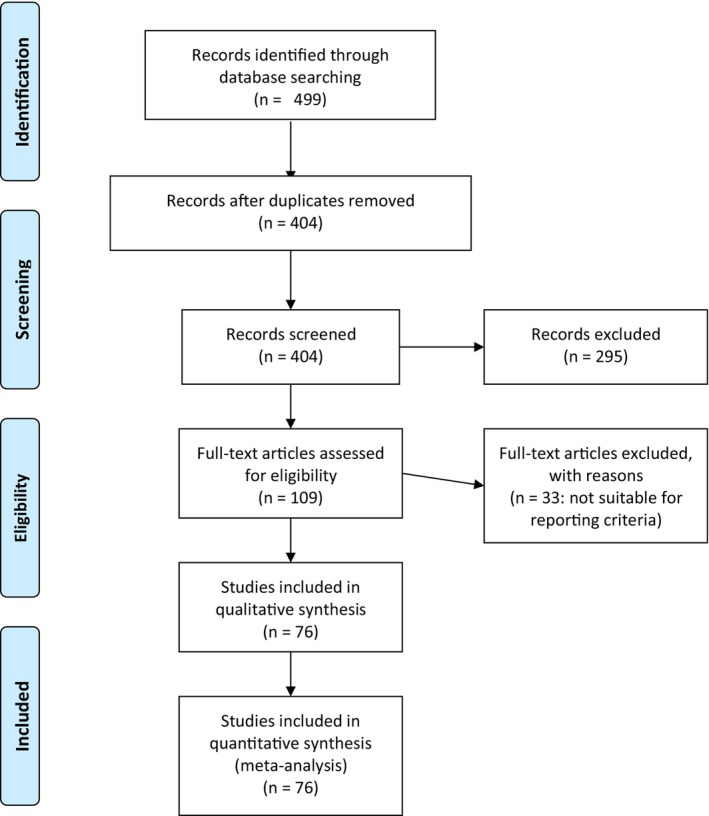
PRISMA flow diagram for systematic review.

We identified a total of 499 articles through database searching. After duplicates were removed, we analyzed titles and abstracts of 404 publications and 109 articles were considered for full‐text evaluation. Seventy‐six studies, 67 case reports, and nine case series (from 29 different countries), were finally included for quantitative analysis (Fig. 3).^4^, ^6^, ^7^, ^14^, ^16^, ^22^, ^23^, ^24^, ^25^, ^26^, ^27^, ^28^, ^29^, ^30^, ^31^, ^32^, ^33^, ^34^, ^35^, ^36^, ^37^, ^38^, ^39^, ^40^, ^41^, ^42^, ^43^, ^44^, ^45^, ^46^, ^47^, ^48^, ^49^, ^50^, ^51^, ^52^, ^53^, ^54^, ^55^, ^56^, ^57^, ^58^, ^59^, ^60^, ^61^, ^62^, ^63^, ^64^, ^65^, ^66^, ^67^, ^68^, ^69^, ^70^, ^71^, ^72^, ^73^, ^74^, ^75^, ^76^, ^77^, ^78^, ^79^, ^80^, ^81^, ^82^, ^83^, ^84^, ^85^, ^86^, ^87^, ^88^, ^89^, ^90^, ^91^, ^92^ Data related to 106 patients who were treated for BRBNS were then extracted.

The baseline characteristics of the overall population included are summarized in Table 1. Briefly, the population analyzed included mainly pediatric patients (i.e. aged under 18 years old, 57.5%) with a slight prevalence of female patients (60.3%). The most frequently affected GI tract was the small bowel, with a peak prevalence of 73.5% of ileal involvement, and half of the patients had already received a treatment for GI bleeding. The most common clinical presentation was melena (53.3%), followed by iron deficiency anemia (26.6%), suggesting an existing biological variance in the manifestation of GI bleeding.

**Table 1:** Baseline characteristics of patients with bleeding blue rubber bleb nevus syndrome

Baseline characteristics	Patient number = 106
Age (median, years)	14.0 (IQR 8.0–20.0)
Female	64 (60.3)
Patients aged under 18 years old	61 (57.5)
Clinical presentation^†^	
Melena	40 (53.3)
Iron deficiency anemia	20 (26.6)
Nonspecified overt bleeding	7 (9.3)
Proctorrhagia	5 (6.6)
Hematemesis	3 (4)
Affected GI tract^‡^	
Esophagus	3 (3.6)
Stomach	42 (50.6)
Duodenum	57 (68.6)
Jejunum	57 (68.6)
Ileum	61 (73.5)
Colon	52 (62.6)
Rectum	5 (6.9)
Previous treatment	
Any treatment	53 (50.0)
Endoscopy	29 (27.3)
Surgery	20 (18.8)
Drugs	11 (10.4)

^†^ Available in 75 cases.

^‡^ Available in 83 cases.

Data are expressed as *n* (%) unless otherwise indicated.

GI, gastrointestinal; IQR, interquartile range.

The patients were divided into three main macro areas of treatment, namely: endoscopy, surgery, and drug treatment; different techniques and/or therapies belonging to the three macro areas of treatment are reported in Table 2.

**Table 2:** Specific treatments for bleeding blue rubber bleb nevus syndrome

	*n* (%)
Endoscopy procedures, *n* = 37	
Single treatment	
Snare mucosectomy	8 (21.6)
Polidocanol injection	6 (16.2)
Sclerotherapy not specified	5 (13.5)
Endoloop	2 (5.4)
Thermal hemostasis	3 (8.1)
Alcohol injection	1 (2.7)
APC	6 (16.2)
ESD	1 (2.7)
Combined therapy	
APC + polidocanol	1 (2.7)
Banding + endoloop	1 (2.7)
Banding + snare mucosectomy	1 (2.7)
Sclerotherapy + snare mucosectomy + endoloop	1 (2.7)
Thermal hemostasis + snare mucosectomy	1 (2.7)
Surgery procedures, *n* = 29	
SB resection	9 (31.0)
SB resection + SB wedge excision	3 (10.3)
SB wedge excision + colon wedge resection	2 (6.9)
Intraoperative enteroscopy thermal hemostasis + surgical SB resection	2 (6.9)
Colon resection	1 (3.4)
Colon resection + SB resection + SB wedge excision	1 (3.4)
Gastrotomy + SB resection + colotomy	1 (3.4)
Gastrotomy + SB resection + SB wedge resection	1 (3.4)
Hemorrhoidectomy	1 (3.4)
Intraoperative enteroscopic snare mucosectomy	1 (3.4)
Proctocolectomy	1 (3.4)
SB resection + intraoperative enteroscopy with snare mucosectomy	1 (3.4)
SB resection + SB wedge excision + endoloop	1 (3.4)
SB resection + sclerotherapy + endoloop	1 (3.4)
SB wedge excision + endoloop	1 (3.4)
SB wedge excision + thermal hemostasis	1 (3.4)
Wedge excision	1 (3.4)
Systemic drug treatment, *n* = 40	
Sirolimus	32 (80.0)
IFN‐alfa + steroids	2 (5.0)
Methlprednisolone	2 (5.0)
Octreotide	2 (5.0)
Everolimus	1 (2.5)
Thalidomide	1 (2.5)

APC, argon plasma coagulation; ESD, endoscopic submucosal dissection; SB, small bowel.

### Endoscopic treatments

Thirty‐seven endoscopic techniques were almost equally distributed between resection and banding/looping techniques (37.8%), hemostatic coagulation (29.7%), and sclerotizing agents (35.1%).

Up to 13.5% of the endoscopic treatments involved two combined techniques (Table 2). When resection, banding, and looping treated cases were analyzed, mucosectomy was the most widely adopted treatment, in 11/15 patients (73.3%), sometimes in association with other techniques (banding = 1, sclerotherapy + endoloop = 1, thermal hemostasis = 1). Only one case of endoscopic submucosal dissection (ESD) has been reported for BRBNS. Endoloop has been reported as stand‐alone endoscopic treatment in two cases, whereas banding was always associated with at least another treatment. Overall clinical success of resection, banding, and looping was reached in 13/15 patients (86.6%). In particular, the only ESD performed was not successful (0/1, 0%) and mucosectomy was successful in 7/8 patients when applied as a stand‐alone treatment (87.5%).

When sclerotizing techniques were adopted, different agents were used (alcohol = 1, polidocanol = 7), although in 5/13 cases the sclerotizing agent was not specified. Two patients were not treated effectively with sclerotizing techniques, namely, one case of unspecified sclerotherapy and one case of polidocanol injection (2/13, 15.3%).

When hemostatic techniques were applied, thermal hemostasis and argon plasma coagulation have been described as stand‐alone treatment in nine patients but have been otherwise employed in association with other endoscopic techniques in another two cases. A case of unsuccessful treatment was disclosed in a patient who was treated with thermal hemostasis only (1/11, 9.0%).

### Surgical treatments

Patients who underwent surgical procedures were mostly treated with entire or wedge resection of the affected GI, and up to one‐quarter of the interventions were assisted by intraoperative enteroscopy (Table S1).

In particular, extensive surgical resections were described in 65.5% patients, whereas 34.5% patients were treated exclusively with a localized technique, such as wedge resections (80%), endoloop application (20%), and thermal hemostasis (30%) during intraoperative endoscopy.

Clinical success was reported in all patients undergoing surgery for BRBNS.

### Systemic drug treatments

The mTOR inhibitors (e.g. sirolimus, everolimus) were the most used drug therapy (82.5%), with sirolimus accounting for most cases (32/33). Clinical success is reported for all the patients treated with sirolimus (100%), whereas clinical improvement was not observed in three patients treated with everolimus (1/1, 100%), thalidomide (1/1, 100%), or octreotide (1/2, 50%).

### Statistical analyses: baseline characteristics and outcomes

When descriptive statistics were applied to our data (Table 3), the three main macro areas of treatment (i.e. endoscopic treatment, surgical operations, systemic drugs) were comparable in terms of age, age at first symptoms, and female‐to‐male ratio. Nearly all the patients had a successful clinical response to therapy, with recurrence occurring in up to one‐quarter of patients, an almost null rate of death due to the diseases, and all these numbers were similar between the three cohorts.

**Table 3:** Difference in reporting between clinical baseline variables and outcomes in the three main treatment choices for bleeding blue rubber bleb nevus syndrome

	Endoscopy (E) *n* = 37	Surgery (S) *n* = 29	Drugs (D) *n* = 40	*P*‐value
Age (median, years)	16.5 (IQR 8.5–21.5)	15.0 (IQR 11.0–22.0)	11.5 (IQR 6.0–18.0)	0.14
Age at first symptoms (median, years)	10.0 (IQR 4.0–15.5)	10.0 (IQR 5.75–13.25)	5.0 (IQR 3.0–11.0)	0.12
Female (%)	18/37 (48.6)	23/29 (79.3)	23/40 (57.5)	E vs. S = 0.06 E vs. D = 0.58 S vs. D = 0.15
Pediatric patients (%)	17/37 (45.9)	13/29 (44.8)	14/40 (35.0)	E vs. S = 1.00 E vs. D = 0.85 S vs. D = 0.85
Previous treatment (%)	12/37 (32.4)	14/29 (48.3)	27/40 (67.5)	E vs. S = 0.29 **E vs. D = 0.01** S vs. D = 0.26
Clinical success (%)	32/37 (86.4)	29/29 (100.0)	37/40 (92.5)	E vs. S = 0.33 E vs. D = 0.62 S vs. D = 0.54
Follow‐up time (median, months)	12.0 (IQR 6.0–28.5)	24.0 (IQR 12.0–45.0)	16.0 (IQR 12.0–21.5)	0.19
Recurrence (%)	6/37 (16.2)	2/29 (6.7)	7/40 (17.5)	E vs. S = 0.66 E vs. D = 1.00 S vs. D = 0.66
Adverse event (%)	4/39 (10.1)	2/27 (7.4)	18/40 (45.0)	E vs. S = 0.91 **E vs. D = <0.01** **S vs. D = <0.01**
Death (%)	0/39 (0.0)	0/29 (0.0)	1/40 (2.5)	E vs. S = NA E vs. D = 1.00 S vs. D = 1.00
Session needed (median)	2 (IQR 1–4)	1 (IQR 1–1)	NA	**<0.01**
Emergency setting (%)	7/30 (23.3)	6/29 (20.6)	NA	0.89

Bold text indicates statistically significant results (*P* < 0.05).

IQR, interquartile range; NA, not applicable.

When applying multiple comparison tests, we found a statistically significant difference between the cohort of patients treated with systemic drugs versus endoscopy in terms of patients who underwent at least one previous treatment (*P* = 0.01). In other terms, this means that if we looked up in the existing literature what has been prescribed to patients who had a symptomatic recurrence of BRBNS lesions, articles reporting systemic drug therapy are more frequent than those reporting endoscopic‐driven treatment. Also, a significant difference in terms of side‐effect was found in patients who received a drug treatment (43.6%) compared to surgical (7.1%) or endoscopic treatment (12.1%) (*P* < 0.01). Notably, mucositis due to mTOR inhibitors administration is the main AE reported in the drug therapy cohort (88.2%). Additionally, when comparing the interventional group of endoscopy and surgery, a higher number of sessions is required (2 vs. 1) in the endoscopic group to achieve clinical success (*P* < 0.01). Besides, no differences were found in terms of cases reported during an emergency scenario (i.e. treated urgently for hemodynamic instability and/or during admission in accident/emergency) between endoscopy and surgery.

## DISCUSSION

Blue rubber bleb nevus syndrome is a rare disease, and this is reflected by the fact that, to the best of our knowledge, only case reports or limited case series have been reported so far. For this reason, we conducted a systematic review to gather evidence from the existing body of the literature, to provide an updated overview and comparison of the effectiveness of different therapeutical options, including medical therapy, endoscopy, and surgical treatments for patients affected by BRBNS.

Our study confirms that in the majority of cases, BRBNS is diagnosed in pediatric patients, and usually involves the small bowel. For this reason, small bowel endoscopy, including capsule endoscopy and device‐assisted enteroscopy, must be considered when investigating patients with suspected BRBNS incidentally found during diagnostic endoscopy or at dermatological examination. Therefore, this category of patients should be referred to small bowel tertiary referral centers with available device‐assisted enteroscopy.

It appears that oral drug treatments (mostly sirolimus) were more frequently administered as second‐line therapy in patients who failed to respond to a different initial treatment, preferring the medical therapy to further endoscopic approaches. Considering our process of indirect evidence gathering, this might be justified by several hypotheses, assuming a strong positivity and publication bias. One possibility is that endoscopic techniques at the moment of recurrence fail to achieve a complete remission. Another explanation could be that the effectiveness of endoscopic techniques at the moment of recurrence is not astounding; therefore, those cases were not considered for publication. Finally, it is possible that the physician in charge of the patients did not believe in a significant impact of an endoscopic treatment on recurrence. However, we cannot rule out that these results happened only by chance and further studies are therefore needed.

Our findings also suggest that medical therapy should be preferred in patients with rebleeding caused by multiple lesions located in different parts of the GI tract, in which endoscopic or surgical treatment might be limited in the longer term, with recurrent bleedings. Although mTOR inhibitor treatment appears to be promising in these patients, their use is partially counterbalanced by AEs. To date, in the only prospective cohort study available on the treatment of BRBNS, the authors enrolled 11 patients affected by BRBNS and prospectively treated them with sirolimus (adjusted to maintain through concentration of 3–10 ng/mL), achieving a significant size reduction of the VMs;^22^ this led to a resolution of the GI bleeding and the anemia in almost all the cases (10/11), with subsequent cessation of the need for blood transfusion and improvement of quality of life. On the other hand, the patients involved in this study experienced some mild self‐limiting AEs, such as mucositis (81.8%), acne (27.3%), and elevated liver enzymes (18.2%).^22^

We also showed that overall clinical success was achieved in 92% (98/106); only one case of death was reported in a pediatric patient due to failure to control the disease with subsequent massive GI hemorrhage.^23^ This optimistic result must be taken with caution, as it entails positivity and publication bias. In fact, we expect articles reporting a positive outcome to be more commonly published, introducing a skewness in the evidence.

BRBNS remains a difficult entity to properly ascertain due to its rarity and its scattered geographical distribution. Due to the lack of prospective multicenter trials and high‐quality evidence, it is difficult to propose a shared treatment algorithm. For example, invasive treatments (i.e. endoscopy and surgery) as a first‐line approach, with sirolimus as a second‐line treatment, can be considered based on concerns over sirolimus life‐long treatment and its side‐effects, which may significantly impact the quality of life. On the opposite, a sirolimus‐based first‐line therapy could be suggested in those patients with countless VMs or when there are concerns over the invasiveness of surgical or endoscopic techniques.^24^

The main limitation of our study is the already discussed nature of the articles analyzed (i.e. case series and case reports). In this sense, all the statistically significant differences we have noted in terms of clinical variables and outcomes should not be considered as real existing differences between treatments. That could only be evident with retrospective cohort studies or (even better) with randomized controlled trials. Instead, our results are quantitative differences in reporting, and we drew conclusions by indirect demonstration. Another potential limitation is that, due to data paucity, we did not compare different kinds of techniques in the same macro area of treatments (e.g. loop technique vs. argon plasma coagulation). As this systematic review relies on case reports and case series coming from centers with different expertise, the results should be considered with a very low/low grade of evidence.

## CONCLUSION

Endoscopy, surgery, and systemic drug therapy are feasible treatment options for BRBNS, but the best treatment options and therapy algorithms are not known yet. Systemic drug therapy was the favorite second‐line treatment after endoscopic failure or recurrence of BRBNS, but AEs were more frequently reported. Therefore, prospective and multicenter studies are indeed warranted, including longer follow‐up time, to confirm the best treatment options for patients with BRBNS.

## CONFLICT OF INTEREST

Authors declare no conflict of interest for this article.

## FUNDING INFORMATION

This study was partially funded by the Italian Ministry of Health – current research given to IRCCS Foundation Ca' Granda Policlinico Milano to finance ordinary research.

## Supporting information


**Table S1** Surgery treatments available for blue rubber bleb nevus syndrome for each gastrointestinal site.
