# Supplementary material for: Treatment options for gastrointestinal bleeding blue rubber bleb nevus syndrome: Systematic review
Source: Dig Endosc. 2023 May 9;36(2):162–71. doi: 10.1111/den.14564 (PMC12136259; doi:10.1111/den.14564)
Supplement: Supplementary file 1 — Table S1 Surgery treatments available for blue rubber bleb nevus syndrome for each gastrointestinal site. [file DEN-36-162-s001.docx]

| **Surgery procedures n = 29** | **No. (%)** |
| --- | --- |
| Small bowel surgery (n = 25)   - Bowel segment resection - Wedge excision - Intraoperative enteroscopy (thermal haemostasis, sclerotherapy, endoloop)   Colon surgery (n = 7)   - Colectomy - Colon resection - Colon wedge excision - Haemorrhoidectomy   Gastric surgery (n = 2)   - Gastrotomy | 20 (80.0)  10 (40.0)  7 (28.0)  2 (28.5)  2 (28.5)  2 (28.5)  1 (14.3)  2 (100.0) |

**Supplementary table 1** – surgery treatments available for BRBNS for each GI site
